# KMT2A regulates cervical cancer cell growth through targeting VDAC1

**DOI:** 10.18632/aging.103229

**Published:** 2020-05-21

**Authors:** Changlin Zhang, Yijun Hua, Huijuan Qiu, Tianze Liu, Qian Long, Wei Liao, Jiehong Qiu, Nang Wang, Miao Chen, Dingbo Shi, Yue Yan, Chuanbo Xie, Wuguo Deng, Tian Li, Yizhuo Li

**Affiliations:** 1 Department of Gynecology, The Seventh Affiliated Hospital of Sun Yat-Sen University, Shenzhen, China; 2 Sun Yat-Sen University Cancer Center, State Key Laboratory of Oncology in South China, Collaborative Innovation Center of Cancer Medicine, Guangzhou, China; 3 The Fifth Affiliated Hospital, Sun Yat-Sen University, Zhuhai, China; 4 College of Life Sciences, Jiaying University, Meizhou, Guangdong, China

**Keywords:** KMT2A, VDAC1, cervical cancer

## Abstract

Cervical cancer is an aggressive cutaneous malignancy, illuminating the molecular mechanisms of tumorigenesis and discovering novel therapeutic targets are urgently needed. KMT2A is a transcriptional co-activator regulating gene expression during early development and hematopoiesis, but the role of KMT2A in cervical cancer remains unknown. Here, we demonstrated that KMT2A regulated cervical cancer growth via targeting VADC1. Knockdown of KMT2A significantly suppressed cell proliferation and migration and induced apoptosis in cervical cancer cells, accompanying with activation of PARP/caspase pathway and inhibition of VADC1. Overexpression of VDAC1 reversed the KMT2A knockdown-mediated regulation of cell proliferation, migration and apoptosis. The *in vivo* results from a cervical cancer xenograft mouse model also validated that KMT2A knockdown suppressed tumor growth by inhibiting VDAC1, whereas KMT2A overexpression promoted cervical cancer growth. Moreover, analyses of Biewenga cervix database and clinical samples showed that both KMT2A and VDAC1 were upregulated in cervix squamous cell carcinoma compared with cervix uteri tissues, and their expression was negatively correlated with the differentiation grade of cervical cancer. Our results therefore indicated that the KMT2A/VDAC1 signaling axis may be a potential new mechanism of cervical carcinogenesis.

## INTRODUCTION

Cervical cancer is a major gynecological cancer that involves uncontrolled cell division and tissue invasiveness of the female uterine cervix [1]. In the past decades, much progress has been made in prevention and treatment in cervical cancer due to early screening [2]; early diagnosis and neoadjuvant chemotherapy [3]. The survival rate of cervical cancer patients has largely improved. In addition, increasing knowledge of the pathophysiology of cervical cancer and a better understanding of the role of the HPV (human papillomavirus) infection in pre-invasive and invasive cervical cancer have led to the development and application of vaccines that targeting cervical cancer [4–6]. Although HPV infection [7] has been found in most of the cervical cancer cases [8, 9], additional genetic (clustered genomic hot spots and a potential microhomology-mediated integration mechanism in cervical cancer) [10] and epigenetic changes are required for disease progression. Recent advances in the biology of cervical cancer reveal that epigenetic alteration is common in cervical carcinogenesis and metastasis [11]. Epigenetic alteration due to aberrant DNA methylation and histone modification has been extensively studied in cervical cancer.

KMT2A, also known as mixed-lineage leukemia (MLL), is a transcriptional coactivator which regulates gene expression during early development and hematopoiesis [12, 13]. KMT2 family proteins methylate lysine 4 on the histone H3 tail at important regulatory regions in the genome and thereby impart crucial functions through modulating chromatin structures and DNA accessibility [14, 15]. KMT2A is often involved in translocation-associated gene fusion and recurrent chromosomal translocations that lead to the development of acute leukemia [16]. The detection of all possible types of KMT2A gene rearrangements is of key importance for the identification of biological subgroups [17, 18]. Subsequent molecular analysis shows a novel variant form of the previously described KMT2A-FLNA fusion gene, in which the KMT2A intron 9 is fused to the FLNA exon 16 [19]. NUP98 (nucleoporin 98 gene) fusion proteins interact with NSL (non-specific lethal) and MLL1 (mixed lineage leukemia 1) complexes to drive Leukemogenesis are reported [20]. Translocations of the KMT2A gene are commonly associated with high-risk de novo or therapy-associated B-cell and T-cell lymphoblastic leukemia and myeloid neoplasms [21]. Other reports show that KMT2A (MLL)-MLLT1 is rearrangement in blastic plasmacytoid dendritic cell neoplasm. KMT2A rearrangement in fetal liver hematopoietic cells is also reported. The efforts to integrate the molecular mechanisms of KMT2A with its roles in tumorigenesis have led to the development of inhibitors of KMT2A. Targeting MLL1-WDR5 protein-protein interaction (PPI) to inhibit the activity of histone methyltransferase of MLL1 complex is a novel strategy [21–24]. Etoposide, a TOP2 inhibitor, is associated with the development of KMT2A -rearranged infant leukemia. At the same time, as a functional gene, KMT2A cooperates with menin to suppress tumorigenesis in mouse pancreatic islets [25]. KMT2A methyltransferase activity mediates KMT2A-dependent transcription and MLL cell survival [26]. Nevertheless, the role of KMT2A in cancers other than leukemia remains largely unknown.

VDAC1 (voltage dependent anion channel 1), a major component of the outer mitochondrial membrane [27], is found at the crossroads of metabolic and survival pathways [28]. The location of VDAC1 at the outer mitochondrial membrane also enables its interaction with proteins that mediate and regulate the integration of mitochondrial functions with cellular activities [29]. It is involved in the process of mitochondria-mediated apoptosis by mediating the release of apoptotic proteins and interacting with anti-apoptotic proteins [30]. The engagement of VDAC1 in the release of apoptotic proteins involves VDAC1 oligomerization that mediates the release of cytochrome c and AIF to the cytosol, subsequently leading to apoptotic cell death. Apoptosis can also be regulated by VDAC1, serving as an anchor point for mitochondria-interacting proteins [31, 32], such as hexokinase (HK) [33], Bcl2 and Bcl-xL, some of which are also highly expressed in many cancers [34, 35]. VDAC1 mediates interactions between mitochondria and other parts of the cell by transporting anions, cations, ATP, Ca^2+^, and metabolites. VDAC1 plays a key role in apoptosis. It regulates the release of apoptogenic proteins from mitochondria, such as cytochrome c, and interacts with anti-apoptotic proteins. The location of VDAC1 at the outer mitochondrial membrane also enables its interaction with proteins that mediate and regulate the integration of mitochondrial functions with cellular activities. As a transporter of metabolites, VDAC1 contributes to the metabolic phenotype of cancer cells [36]. It is over-expressed in many cancer types, and silencing of VDAC1 expression induces an inhibition of tumor development [37].

In this study, we investigated the role of KMT2A in the regulation of cervical cancer cell growth. We also identified the underlying molecular mechanisms of KMT2A in cervical cancer and its clinical significance. Our results showed that KMT2A regulated cervical cancer growth by targeting VDAC1 signaling. In addition, we also analyzed the expression levels of KMT2A and VADC1 in online databases and cervical cancer patients and evaluated their clinical implications. Our study indicates the KMT2A/VADC1 signaling axis may be a new mechanism of cervical tumorigenesis and a potential therapeutic target for cervical cancer treatment.

## RESULTS

### KMT2A knockdown suppressed cell proliferation, migration and induced apoptosis in cervical cancer cells

**Figure 1:**
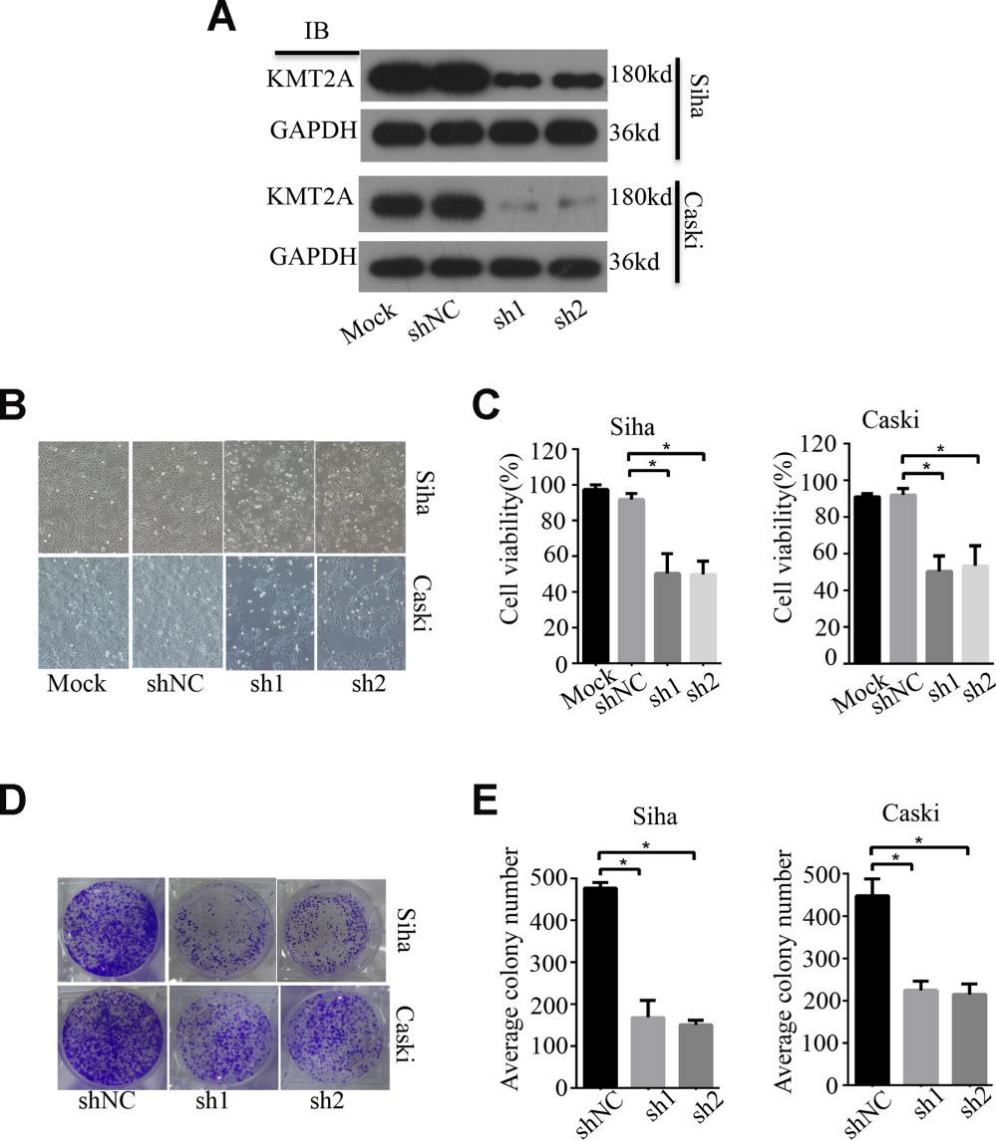
**KMT2A Knockdown suppressed cell viability and colony formation in cervical cancer cells.** (**A**) The protein levels of KMT2A in Siha and Caski cells were detected by Western blot. (**B**) The viability of Siha and Caski cells was measured by MTS assay. (**C**) The average cell viability %. (**D**) Colony formation of Siha and Caski cells. (**E**) The average value of colony number.

To examine the biological function of KMT2A in cervical cancer, we measured its effect on cell growth in human cervical cancer cell lines by MTS and colony formation assays. KMT2A knockdown by its specific shRNAs (sh1 and sh2) dramatically inhibited the expression of KMT2A protein in SiHa and Caski cells (Figure 1A), and significantly suppressed cell viability (Figure 1B, 1C) and resulted in a marked reduction in the number of the colony in cervical cancer cells (Figure 1D, 1E).

**Figure 2:**
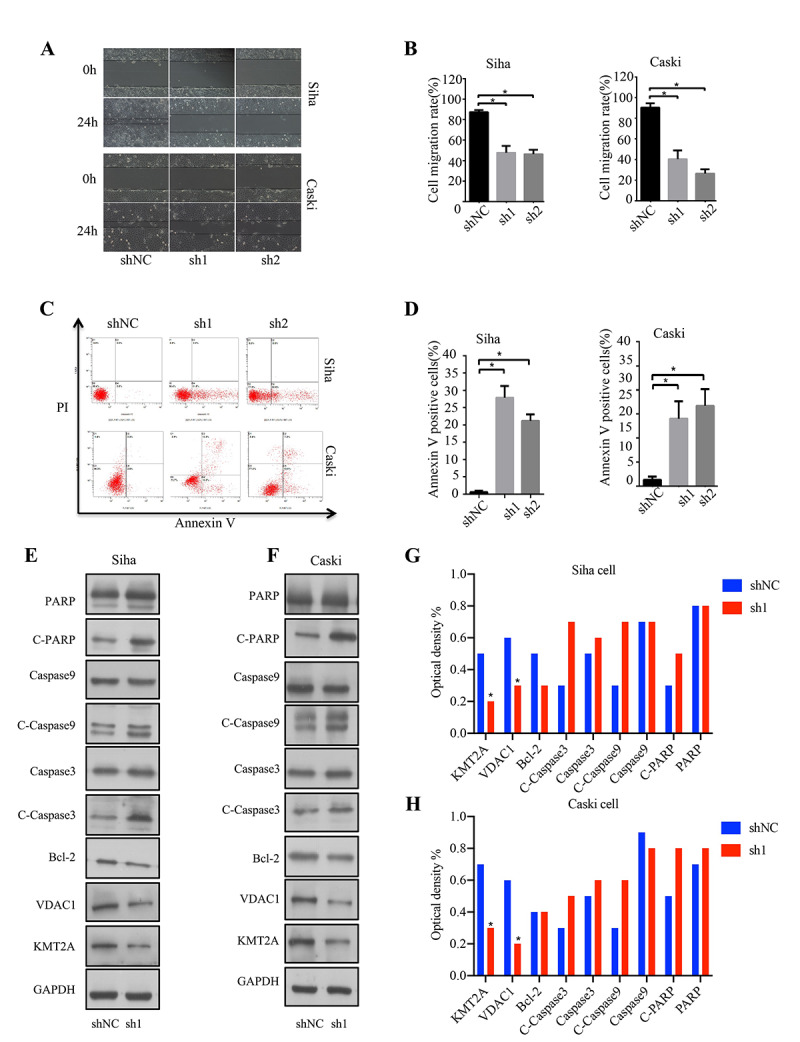
**KMT2A knockdown suppressed cell migration and induced apoptosis in cervical cancer cells.** (**A**) The migration ability of Siha and Caski cells was measured by wound-healing assay. (**B**) The cell migration rate %. (**C**) Apoptosis of Siha and Caski cells was detected by FACS analysis. (**D**) Annexin V positive cells %. (**E** and **F**) The expression of proteins was detected by Western blot in Siha and Caski cells with KMT2A knockdown. (**G** and **H**) The average value of Figure **E** and **F**.

In addition, we also evaluated the effect of KMT2A knockdown on cell migration in SiHa and Caski cells by wound-healing assay. At 48 hours after cell scratching, the width of the gap or wounding space between cell layers remained distinct in the cells treated with KMT2A specific shRNAs, but the gap was almost fully occupied by the migrating cells in the control group (Figure 2A, 2B). We further determined whether the KMT2A knockdown mediated inhibition of cell growth was associated with apoptosis. The SiHa and Caski cells were transfected with KMT2A specific or scrabbled control shRNAs for 48 hours and subjected to apoptosis analysis by FACS. We found that KMT2A knockdown induced a higher percentage of apoptotic cells compared to the control shRNA (Figure 2C, 2D).

To identify the underlying molecular mechanism of KMT2A in regulating cervical cancer cell growth, we analyzed the effect of KMT2A on the expression of a series of apoptosis-related proteins. As shown in Figure 2E and 2F, KMT2A knockdown in SiHa and Caski cells dramatically inhibited VDAC1 protein expression, and increased the levels of cleaved-caspase 3, cleaved-caspase 9 and cleaved-PARP. These results indicated that KMT2A regulated cell growth and apoptosis via targeting VDAC1 and caspase-dependent signaling pathways. Considering that VADC1 can mediate the release of apoptotic proteins and interacts with anti-apoptotic proteins and plays a key role in the process of mitochondria-mediated apoptosis, in this study we are interested in and focused on the KMT2A/VDAC1 signaling axis in cervical cancer.

### VDAC1 and KMT2A were upregulated in cervical cancer and negatively correlated with differentiation grade

**Figure 3:**
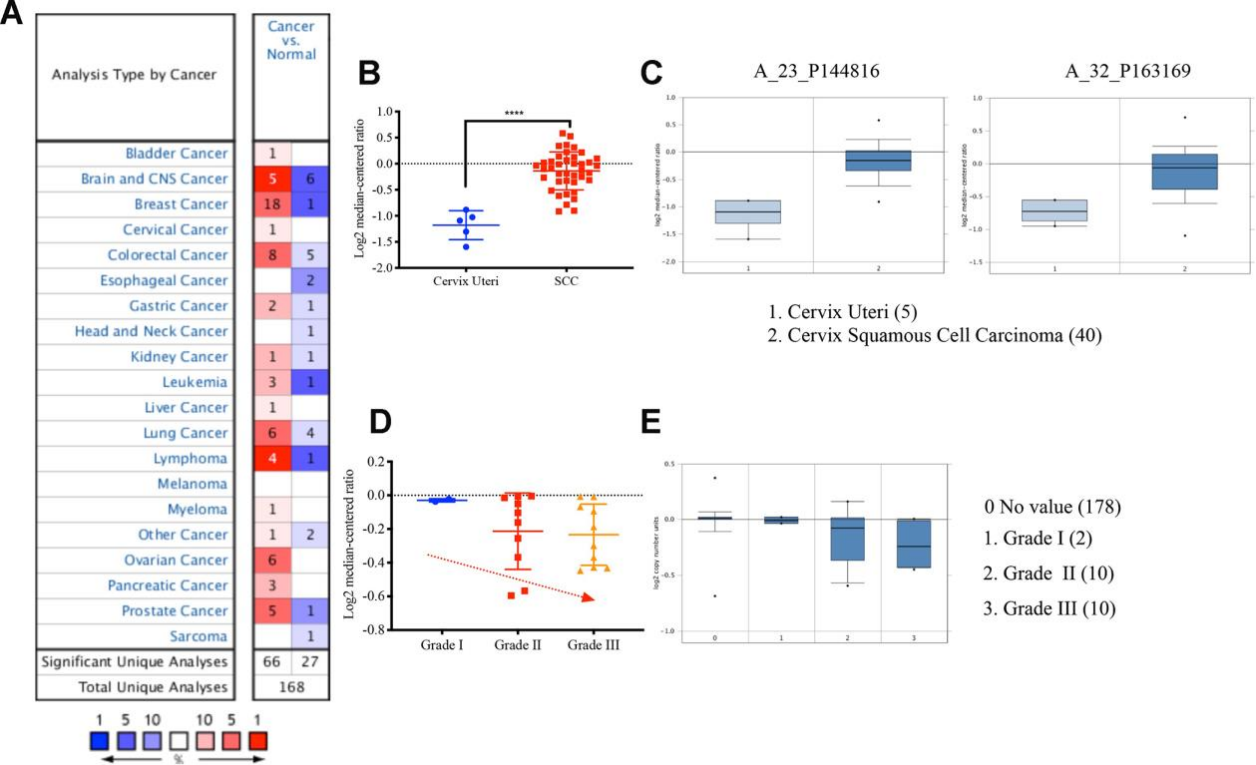
**The VDAC1 mRNA was upregulated in cervical cancer by analyzing Oncomine data.** (**A**) Upregulation of VDAC1 in one of six databases. (**B**) The expression of VDAC1 mRNA in cervical squamous cell carcinoma and cervix uteri. (**C**) Level of VDAC1 mRNA in cervix uteri and cervical squamous cell carcinoma in two probes (A_23_P144816, A_32_P163169) of Biewenga Cervix database. (**D** and **E**) The relationship between the expression of VDAC1 and the differentiation grade of cervical cancer. The red color represents increase. The deeper the red is, the greater the increase. The blue represents decrease. The deeper the blue is, the greater the decrease.

We next analyzed the expression level of VDAC1 and its clinical significance in cervical cancer using Oncomine database. We found that the expression of VDAC1 in cervical cancer was higher than the cervix normal tissue in one of six databases (Figure 3A), and the expression of VDAC1 was upregulated in Biewenga Cervix database (Figure 3B). Compared with the cervix uteri tissues, VDAC1 was overexpressed in cervix squamous cell carcinoma. As shown in Figure 3C, the results from the different probes in Biewenga Cervix database also verified high expression of VDAC1 in cervix squamous cell carcinoma by comparison with cervix uteri tissues. Moreover, we found a negative correlation between cervical cancer differentiation grade and VADC1 expression. As the grade of cervical cancer deepened, the expression of VDAC1 decreased (Figure 3D, 3E).

In addition, we also analyzed the expression level of KMT2A and its clinical significance in cervical cancer using Oncomine database. As shown in Supplementary Figure 1A, after setting the filter conditions (P<0.0001, fold change>1.5 and the TOP 10% data), there are not many databases which can meet the requirements, and the correlation between KMT2A and different cancers were not very common in Oncomine database. We then searched Biewenga Cervix database and found that the expression of KMT2A was upregulated in cervical squamous cell carcinoma compared with the cervix uteri (Supplementary Figure 1B), and the differentiation grade of cervical cancer was negatively correlated with KMT2A expression (Supplementary Figure 1C).

### KMT2A regulated cervical cancer cell viability and apoptosis by targeting VDAC1

**Figure 4:**
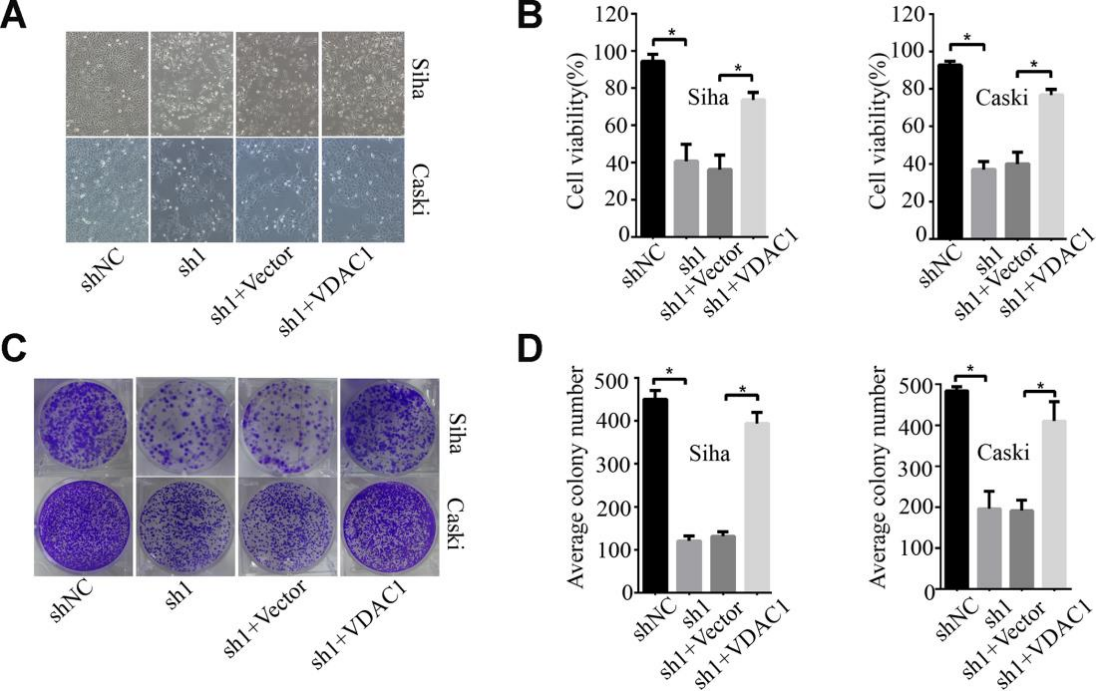
**KMT2A regulated cervical cancer cell proliferation and colony formation by targeting VDAC1.** Human cervical cancer Siha and Caski cells were transfected with KMT2A shRNAs or KMT2A shRNA + VDAC1 overexpression plasmid. At 48 hours after transfection, the cell viability, colony formation and migration ability were measured. (**A**) Viable Siha and Caski cells. (**B**) The average cell viability %. (**C**) Colony formation of Siha and Caski cells. (**D**) The average count numbers.

**Figure 5:**
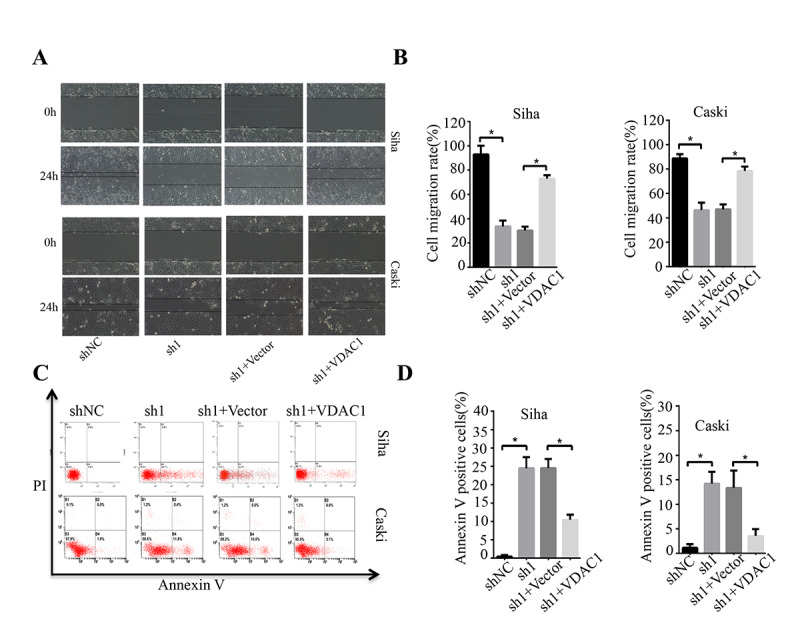
**KMT2A regulated cervical cancer cell migration and apoptosis through targeting VDAC1.** Human cervical cancer Siha and Caski cells were transfected with KMT2A shRNAs or KMT2A shRNA + VDAC1 overexpression plasmid. At 48 hours after transfection, the cell migration and apoptosis rates were measured. (**A**) The migration ability of Siha and Caski cells was measured by wound-healing assay. (**B**) The cell migration rate %. (**C**) Apoptosis of Siha and Caski cells was detected by FACS analysis. (**D**) Annexin V positive cells %.

To determine the possible regulation of KMT2A on VDAC1, we analyzed the effects of KMT2A and VDAC1 on cell growth, migration and apoptosis in cervical cancer cells. Knockdown of KMT2A significantly suppressed cell proliferation (Figure 4A, 4B) and clone formation ability in SiHa and Caski cells (Figure 4C, 4D), whereas VDAC1 overexpression effectively reversed the KMT2A knockdown-mediated suppression of cell viability (Figure 4A, 4B) and clone formation (Figure 4C, 4D). KMT2A knockdown also dramatically inhibited cell migration (Figure 5A, 5B) and induced cell apoptosis (Figure 5C, 5D) in SiHa and Caski cells, while VDAC1 overexpression reversed the KMT2A knockdown-mediated cell migration inhibition (Figure 5A, 5B) and apoptosis induction (Figure 5C, 5D). These results confirmed that KMT2A regulated cervical cancer cell growth through targeting VDAC1 signaling.

### KMT2A knockdown inhibited tumor growth in a cervical cancer mouse xenograft model

**Figure 6:**
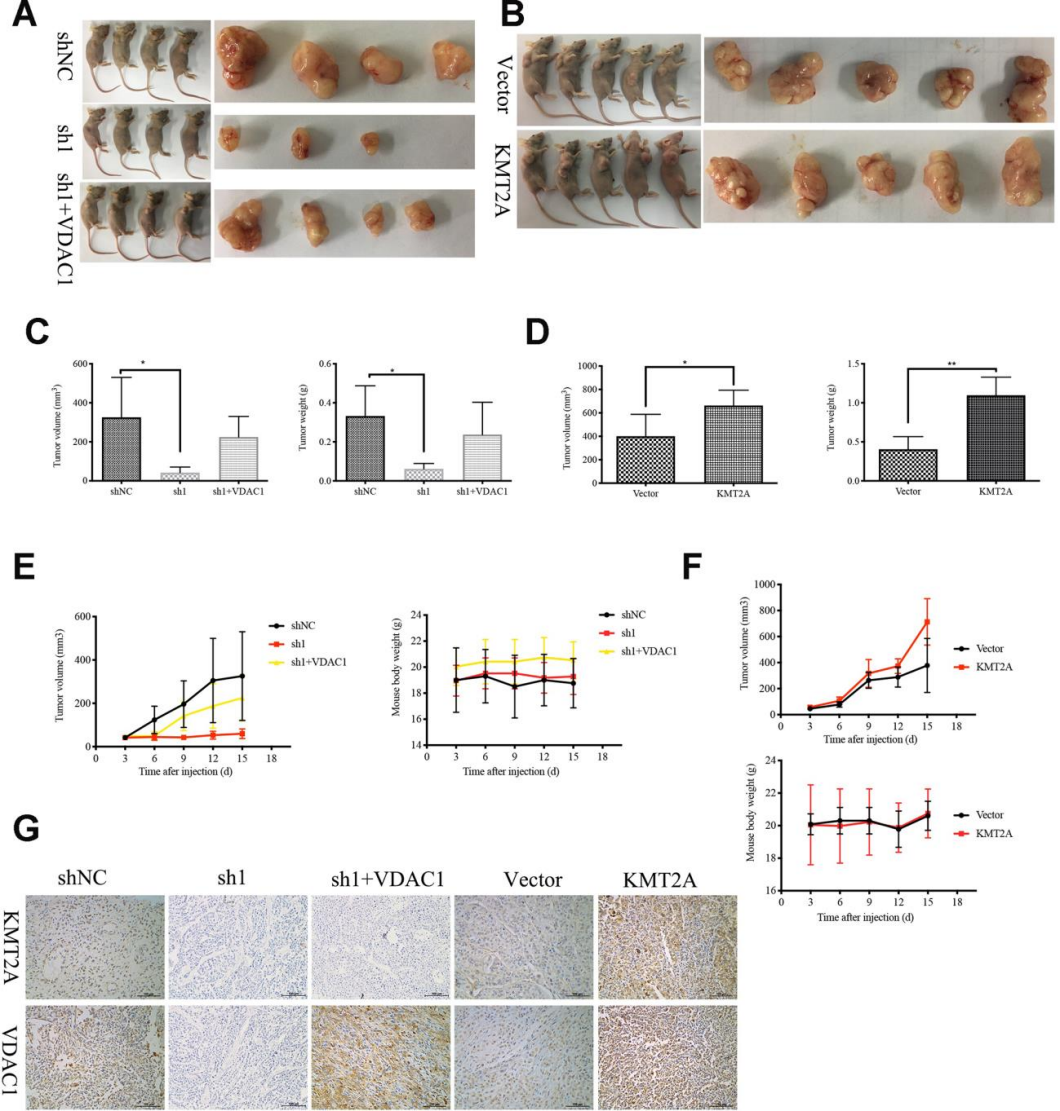
**KMT2A knockdown inhibited cervical cancer growth in a mouse xenograft model.** The control shRNA (shNC), KMT2A-shRNA (sh1), KMT2A-shRNA + VDAC1 overexpression (sh1+VDAC1), empty vector (Vector) and KMT2A overexpression plasmid (KMT2A) were intratumorally injected into mice. (**A** and **B**) Representative photographs of the tumor bearing mice and morphology of tumor xenograft from each mouse. (**C** and **D**) The average tumor volume and the average tumor weight of each group with KMT2A knockdown (**C**) and KMT2A overexpression (**D**). (**E** and **F**) The tumor volume of each mouse was measured and recorded every three days and body weight of each mouse was monitored every three days with KMT2A knockdown (**E**) and KMT2A overexpression (**F**). (**G**) The expression of the proteins in tumor xenografts were tested by immunohistochemical staining. (400× magnification).

We further confirmed the role of KMT2A in regulating cervical cancer growth in a mouse xenograft model. Knockdown of KMT2A dramatically suppressed cervical cancer tumor growth in size, volume and weight (Figure 6A, 6C, 6E), while overexpression of KMT2A promoted tumor growth (Figure 6B, 6D, 6F). Furthermore, VDAC1 overexpression remarkably reversed the growth inhibition caused by KMT2A knockdown (Figure 6A, 6C, 6E). All these treatments did not affect the body weight of the mice (Figure 6E, 6F), and no other signs of acute or delayed toxicity were observed in the mice during treatment.

We also analyzed the efficiency of KMT2A shRNA knockdown and KMT2A overexpression in tumors by detecting the expression of KMT2A and VDAC1 by immunohistochemical (IHC) staining (Figure 6G). Consistent with the in vitro results, knockdown of KMT2A significantly inhibited the expression of KMT2A and VDAC1 proteins, and these inhibitions were reversed by VDAC1 overexpression (Figure 6G).

### KMT2A and VADC1 expression was correlated with cancer type, clinical and N stages in cervical cancer patients

**Figure 7:**
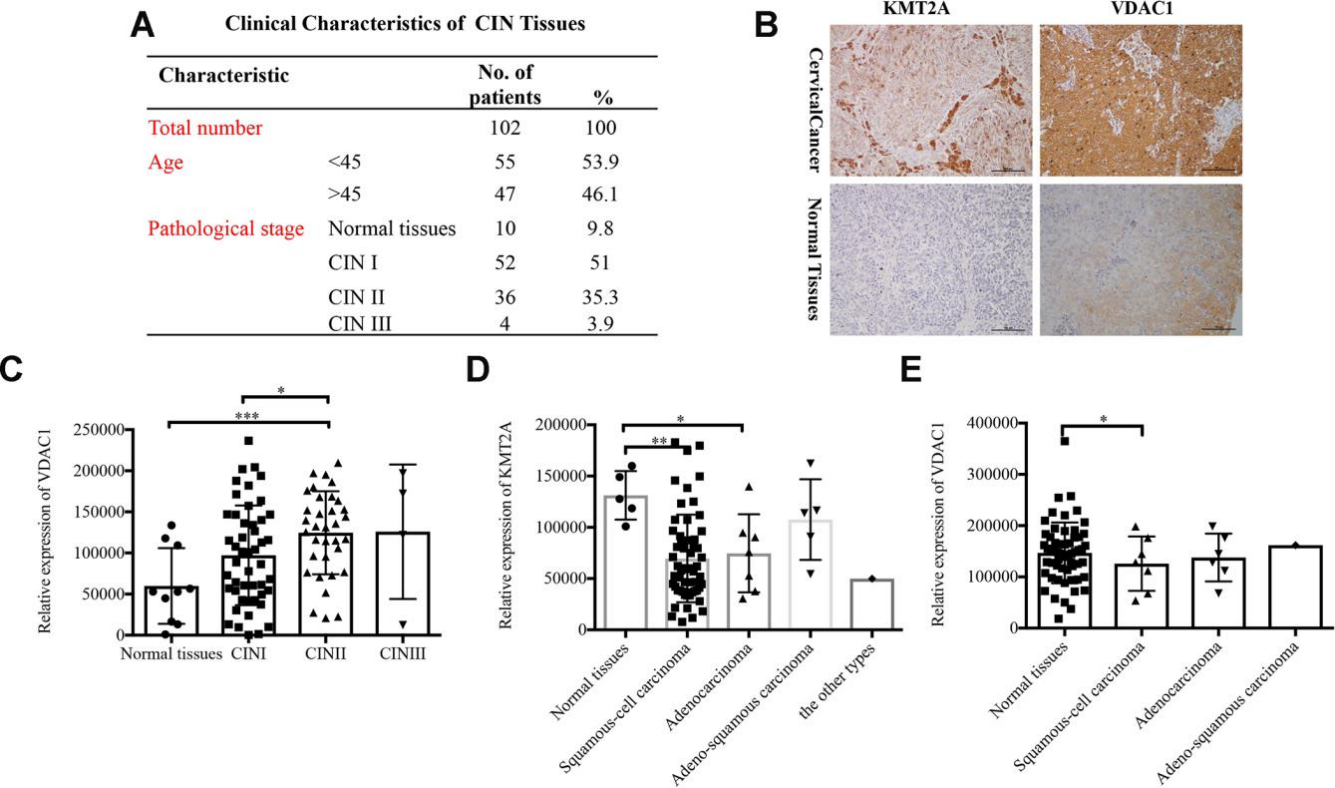
**Expression of KMT2A and VADC1 was significantly correlated with cancer type in cervical cancer patients.** (**A**) The clinical characteristics of cervical intraepithelial neoplasia. (**B**) Representative images of the immunohistochemical staining of KMT2A and VDAC1 in human normal and cervical cancer tissues. 400× magnification. (**C**) The correlation between the expression of VDAC1 and the stage of CIN in human cervical intraepithelial neoplasia tissues from 102 patients. (**D**) The correlation between the expression of KMT2A and different types of cervical cancer in human cervical cancer tissues from 48 patients. (**E**) The correlation between the expression of VDAC1 and different types of cervical cancer in human cervical cancer tissues from 48 patients.

To identify the clinical significance of KMT2A/VDAC1 signaling axis, we detected their expression in cervical intraepithelial neoplasia (CIN) and cervical cancer tissue samples. The clinicopathological data of CIN and representative staining images of KMT2A and VDAC1 in cervical cancer and normal tissues were shown in Figure 7A and 7B. Next, we performed the correlation analysis for these samples and showed that VADC1 expression was positively correlated with CIN stage in cervical cancer patients. As the stage of CIN deepened, the expression level of VADC1 increased (Figure 7C). Moreover, we analyzed the relationships of KMT2A/VDAC1 expression with different clinicopathologic variables, and found that the expression of KMT2A and VDAC1 was significantly correlated with the tumor type (Figure 7D, 7E).

We also analyzed the relationships of different clinicopathologic variables (Supplementary Figure 2C) with KMT2A/VDAC1 expression in cervical cancer patients. We found that there were no significant correlation of KMT2A/VDAC1 expression with patient ages (Supplementary Figure 2A, 2B, 2D, 2H) and cancer grade (Supplementary Figure 2E, 2I). Interestingly, for the clinical stages, when comparing T1, T2, T3/T4 samples, we could see a trend towards high expression of KMT2A and VDAC1 in T3/T4 stage (Supplementary Figure 2F, 2J). For the N stage, when comparing N0 and N1 samples, we could also see a similar trend towards high expression of KMT2A and VDAC1 in the N1 group (Supplementary Figure 2G, 2K).

## DISCUSSION

The structure and biological functions of the KMT2A protein has been deeply investigated in leukemia [38, 39], however, very few studies have explored the functions of KMT2A in malignant cervical cancer. In this study, we have demonstrated the functional significance of KMT2A in cervical cancer progression *in vitro* and *in vivo*. Firstly, we demonstrated that knockdown of KMT2A inhibited cell viability and cell migration and induced cell apoptosis. We also found that a series of apoptosis-related proteins were affected by KMT2A knockdown in cervical cancer cells. Of them, VADC1, a protein on the mitochondrial outer membrane, varied significantly. We analyzed the expression of VDAC1 in Oncomine database and found that it was consistent with the results of our previous research [40]. To investigate the possible mechanism in KMT2A regulating VDAC1in promoting cell growth, we performed the rescued assay and found that overexpression of VDAC1 rescued the cell viability inhibition and cell apoptosis caused by KMT2A knockdown, revealing that VDAC1 signaling was involved in the KMT2A-mediated regulation of cervical cancer viability, cell migration and apoptosis.

We also explored the relationship between KMT2A and VDAC1 and their roles in the regulation of tumor growth in a mouse xenograft model. The similar results were obtained. At the beginning of the experiments, there were 4-5 mice for each group in all groups. Unfortunately, at the end of the experiment, some mice in the groups died, so there were only 3 or 4 mice in some groups. In the future study, we will further investigate the role of KMT2A/VADC1 signaling axis in an orthotopic model.

It has been widely acknowledged that smaller tumor size and early stage of cervical cancer have a more favorable prognosis while metastasis situation has poorer prognosis. In the cervical cancer tissues, the expression of KMT2A was correlation between normal tissues and SCC patients. The differentiation grade of cervical cancer is negatively associated with clinical stage and tumor development and survival. In our study (Supplementary Figure 1), we also showed that the expression levels of KMT2A is negatively associated with the differentiation grade of cervical cancer. However, because the sample number of normal tissues was limited, our analysis showed that KMT2A expression was negatively correlated with CIN stage in cervical cancer patients. In the future research, we will add more samples to test its clinical implications. At the same time, we also checked the status of KMT2A in clinical samples in the Oncomine database. Because there is not much data in the website, we failed to combine the data for conjoint analysis.

Moreover, we found that KMT2A expression is related to the type of clinical cervical cancer. We could see a trend towards high expression of KMT2A and VDAC1 in the T3/T4 stage and in the N1 group. Unfortunately, the difference in both comparisons were not significant. This may be caused by the small sample volumes. In the future study, we will increase sample sizes to solve the problem. To the best of our knowledge, this is the first study to document the role and molecular mechanism of KMT2A in cervical cancer carcinogenesis and development.

KMT2A is a transcriptional coactivator with histone H3 lysine 4 (H3K4) methyltransferase activity [12, 13]. Here, we discovered that KMT2A regulated the cell viability, cell migration and cell apoptosis in cervical cancer, which has been certificated in melanoma and acute lymphoblastic leukemia (ALL). In AML or MLL, chromosomal abnormalities are quite normal and often used as diagnostic, prognostic and predictive biomarkers to provide subtype, outcome and drug response information [41, 42]. Genetic rearrangements of KMT2A result in oncogenic fusion proteins, here, t (17;19)/TCF3-HLF, haploidy or low hypodiploidy are high-risk biomarkers in AML [19]. As the application of genomic technologies to cases without an established abnormality (B-other) reveals copy number alterations, which can be used either individually or in combination as prognostic biomarkers [13]. In cervical cancer, KMT2A revealed its function as a functional gene. Does KMT2A also form a fusion gene to reveal its unique biological function and whether the biological function of KMT2A in cervical cancer cells was related to its methylation transferase activity or not? Such questions need to be investigated in the following assays. Fluorescence in situ hybridization [10] and transcriptase-sequencing studies could give us some keys.

Alterations in cellular metabolism and bioenergetics are vital for cancer cell growth [43]. In the center of the process of energy metabolism, mitochondria are the most important [44]. VDAC1is a master gatekeeper regulating the flux of metabolites and ions between mitochondria and the cytoplasm [45]. In this study, we investigated the role of KMT2A on cervical cancer cell growth, cell migration and cell apoptosis via VDAC1signal and found that VDAC1 was in downstream of KMT2A in the process of cervical cancer cell growth. At the same time, we analyzed the expression level of VDAC1 and its clinical significance in cervical cancer using Oncomine database. We found that the expression of VDAC1 in cervical cancer was higher than the cervix normal tissue in one of six databases. It is better to test the protein levels of VADC1. Regrettably, the data about VADC1 protein is not so much in Oncomine database, so we failed to analyze the protein expression status of VDAC1 in database. In the future research, we will add more samples to analyze the protein expression status of VDAC1 and test its clinical implications. Cervical cancer is positively correlative with HPV infection. Previous study showed that VDAC1 expression was positively related to HPV16 E7 expression in normal tissues and CIN tissues and in HPV16 E7-positive cell lines [40]. Nevertheless, the specific mechanism by which KMT2A targeted VDAC1, thereby regulating cell growth in cervical cancer cells remains to be further explored.

## CONCLUSIONS

In summary, our study has demonstrated that KMT2A regulates cervical cancer cell growth via targeting VDAC1 signaling, indicating that the KMT2A/VADC1 signaling axis may be a new mechanism of cervical tumorigenesis and a potential therapeutic target for cervical cancer treatment.

## MATERIALS AND METHODS

### Cell lines and cell culture

Human cervical cancer cell lines SiHa and Caski were obtained from Procell. SiHa was cultured in MEM with 10% FBS, 100 μg/ml penicillin and 100 μg/ml streptomycin. Caski was cultured in 1640 with 10% FBS, 100 μg/ml penicillin and 100 μg/ml streptomycin and maintained in standard culture condition.

### Reagents and antibodies

GAPDH, VDAC1 antibodies were from Proteintech (Rosemont, IL). KMT2A antibodies were from ABclone, Novus Biological and Santa Cruz Biotechnology (Santa Cruz, CA). PARP, Caspase3, Caspase9, P53, Bcl-2, cleaved-caspase3, cleaved-caspase9, cleaved-PARP, antibodies were from Cell Signaling Technology (Beverly, MA, USA).

### Plasmid vectors

The plasmid pMSCV-Flag MLL-pl-ENL (5613) (KMT2A) and its corresponding vector were gifts from Robert Slany (Addgene plasmid # 20873). The plasmid encoding for VDAC1 was constructed by GeneCreate (Wuhan). The transfection reagent Endo-Fectin^TM^ Max transfection (GeneCopoeia) was used according to the manufacturer’s instructions to transfect human cervical cancer cell lines. The ratio of the reagent to DNA for each cell line was optimized in preliminary experiments. The experiments were performed in triplicate and repeated three times.

### shRNA design

The shRNAs (see Supplementary Table 1 for sequences) were purchased from Shanghai GenePharma Company (Shanghai, China).

### Real time PCR (qPCR)

Total RNA was extracted from cultured cell lines by using RaPure Total RNA Micro kit (Magen Bio) according to the manufacturer’s instructions. cDNA synthesis was performed using HiScript II Q RT SuperMix for qPCR (+ gRNA wiper) (Wazyme R223-01) according to the protocol described, and RT-PCR was finished by using ChamQ^TM^ SYBR qPCR Master Mix (Wazyme R311-02/03) as recommended by the manufacturer, and then followed by detection with a Bio-Rad CFX96 and analyzed with the Bio-Rad Manager software (Bio-Rad, Hercules, CA). The PCR primers were synthesized by shanghai Shenggong (see Supplementary Table 1 for sequences).

### Western blot

Cells were lysed on ice in protein extraction reagent and protein concentration was determined by using Coomassie brilliant blue Assay. Equal amounts of cell extracts were subjected to electrophoresis in 10-15% gradient SDS-PAGE gels, and then transferred to Polyvinylidene Fluoride membranes, and immunoblotted with specific primary antibodies, followed by incubation with HRP-conjugated secondary antibody and finally detected by using ECL (Electro-Chemi-Luminescence) substrates.

### Cell viability assay by MTS

Cells plated in 96-well plates (4000-6000 cells/well) were treated with plasmids or shRNAs. After treatment for the desired time, CellTiter 96® AQueous One Solution Cell Proliferation Assay was used to detect the cell viability. This reagent contains 3-(4,5-dimethylthiazol-2-yl)-5-(3-carboxymethoxyphenyl)-2-(4-sulfophenyl)-2H-tetrazolium and phenazine ethosulfate. 10% MTS reagent was added to the cells with continuous culture for another 2-4 h. The absorbance value at OD490 was detected.

### Colony formation assays

Approximately 500–1,000 cells were seeded into 6-well plates in triplicate and incubated for 10–14 days. Then the cells were washed with PBS and fixed with the mixture (methanol: glacial: acetic=1:1:8) for 10 min, and stained with 0.1% crystal violet for 15 min. After enough washing by PBS, cell colonies that contained more than 50 cells were counted and photographed.

### Wound-healing assay

The wound-healing assay was performed to examine the cellular capability of migration. Cells were plated in a six-well plate and grown to nearly 70-80% confluence. Then the cells were treated with KMT2A shRNAs or plasmids for 12 h and scraped in a straight line to create a “scratch”. The images of the cells at the beginning and at regular intervals during cell migration to close the scratch were captured and compared through quantifying the migration rate of the cells.

### Apoptosis assay

Apoptosis was measured based on FACS analysis using Annexin V PE/7-AAD (Beijing 4A Biotech Co., Ltd, FXP027-050) staining. Cells seeded in a six-well plate were transfected with indicated shRNAs. After 48 h, cells were collected, washed twice with cold PBS, resuspended with cold binding buffer and subsequently stained with AnnexinV-PE and 7-AAD. Stained cells were then analyzed by using FACS.

### Animal experiment and tissue processing

All animal procedures were performed in accordance with the Guide for the Care and Use of Laboratory Animals (NIH publications Nos. 80-23, revised 1996) and the Institutional Ethical Guidelines for Animal Experiments developed by Sun Yat-sen University. SiHa cells (5×10^6^ in 100 μl PBS) were injected subcutaneously into the left flank of female athymic nude mice aged 3-4 weeks. When the formed tumor reached 100 mm^3^, the animals were randomly divided into five groups (4-5 per group) and respectively intratumorally injected with control shRNA, KMT2A shRNA, KMT2A shRNA + VDAC1 overexpression, vector and KMT2A overexpression once every three days for 6 times. The tumor size was measured using a Vernier caliper and the tumor volume was calculated as V = (length × width × height)/2. The experiment was terminated 22 days after tumor cell inoculation. The mice were then sacrificed, and the tumors were excised and weighed. Tumor tissues from the above treated animals were processed as described previous.

### Cervical intraepithelial neoplasia tissue and cervical cancer tissue microarray

Tissue microarray for KMT2A and VDAC1 expression was purchased from Fanpu Biotech, Inc. (Guilin, China), with 102 cervical intraepithelial neoplasia tissues and 75 cervical cancer tissues from patients without anti-cancer treatments. Clinicopathologic information was documented for all cases.

### Immunohistochemical (IHC) assay

Paraffin-embedded tissue specimens were sectioned, deparaffinized in xylene and rehydrated with graded ethanol solution. Antigenic retrieval was then processed with sodium citrate. IHC staining was performed using Streptavidin Peroxidase IHC assay kit (ZSGB-bio, China). The antibodies against KMT2A (Novus Biologicals, dilution 1:50), VDAC1 (Proteintech, dilution 1:200) were used.

### TNM stage

According to the AJCC (American Joint Committee on Cancer) cancer staging manual, the following description means TNM stage. T: primary tumor, T1: cervical carcinoma confined to uterus (extension to corpus should be disregarded), T2: cervical carcinoma invades beyond uterus but not to pelvic wall or to lower third of vagina, T3: tumor extends to pelvic wall and/or involves lower third of vagina, and/or causes hydronephrosis or nonfunctioning kidney, T4: tumor invades mucosa of bladder or rectum, and/or extends beyond true pelvis (bullous edema is not sufficient to classify a tumor as T4), N: regional lymph nodes, NX: regional lymph nodes cannot be assessed, N0: no regional lymph node metastasis, N1: regional lymph node metastasis, M: distant Metastasis, M0: no distant metastasis, M1: distant metastasis (including peritoneal spread, involvement of supraclavicular, mediastinal, or paraaortic lymph nodes, lung, liver, or bone).

### Statistical analysis

Each experiment was done three times and the results were presented as the mean ± SD. GraphPad Prism was used for statistical analysis. Student’s *t*-test was used and *P < 0.05, ***P < 0.001, and ****P < 0.0001 indicated significant difference.

## Supplementary Material

Supplementary Figures

Supplementary Table 1
