# Supplementary material for: KMT2A regulates cervical cancer cell growth through targeting VDAC1
Source: Aging (Albany NY). 2020 May 21;12(10):9604–20. doi: 10.18632/aging.103229 (PMC7288919; doi:10.18632/aging.103229)
Supplement: Supplementary Figures [file aging-12-103229-s001..pdf]

SUPPLEMENTARY FIGURES

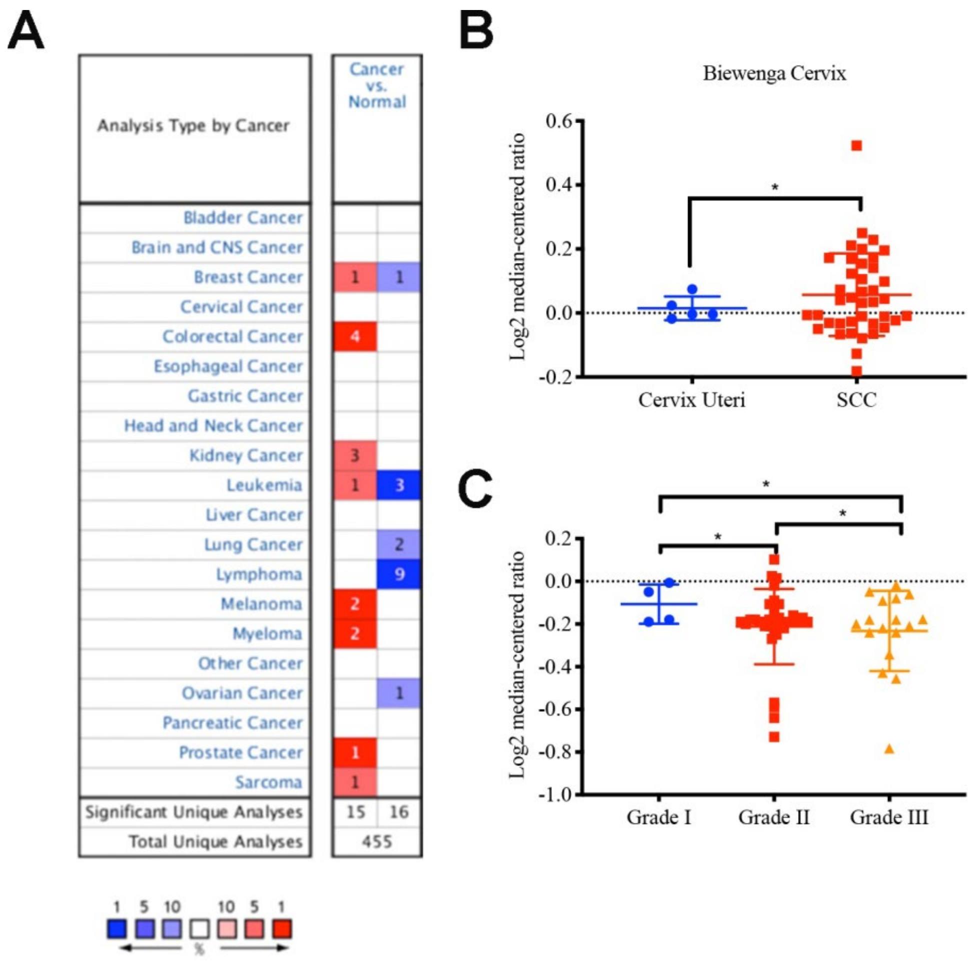

Supplementary Figure 1. The KMT2A mRNA is upregulated in cervical cancer by analyzing Oncomine data. (**A**) The expression profile of KMT2A in different cancers in Oncomine data. (**B**) The expression of KMT2A mRNA in cervical squamous cell carcinoma and cervix uteri in Biewenga Cervix database. (**C**) The relationship of KMT2A mRNA and the differentiation grade of cervical cancer and cervix uteri in Biewenga Cervix database. The red color represents increase. The deeper the red is, the greater the increase. The blue represents decrease. The deeper the blue is, the greater the decrease.

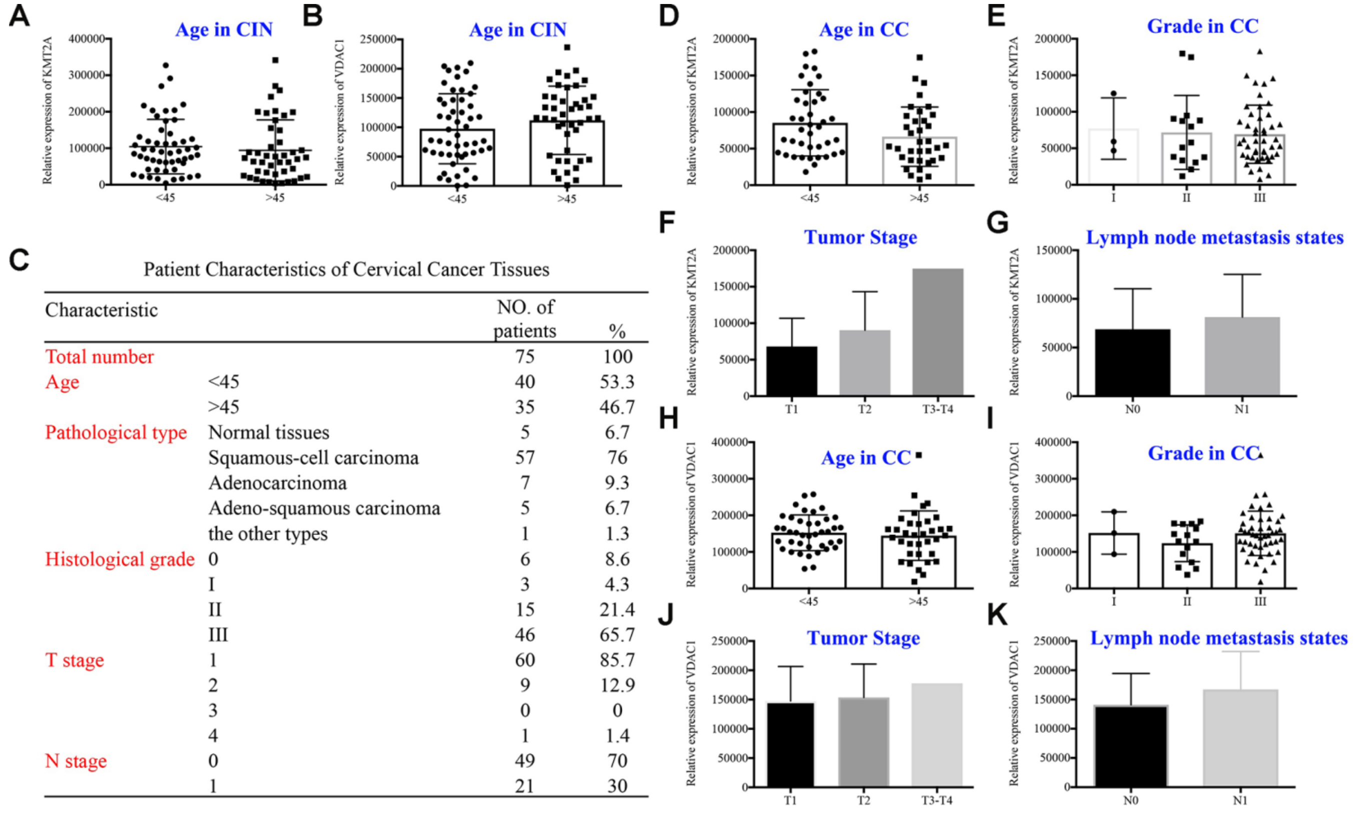

Patient Characteristics of Cervical Cancer Tissues

Characteristic		NO. of patients	%
Total number		75	100
Age	<45	40	53.3
	>45	35	46.7
Pathological type	Normal tissues	5	6.7
	Squamous-cell carcinoma	57	76
	Adenocarcinoma	7	9.3
	Adeno-squamous carcinoma	5	6.7
	the other types	1	1.3
Histological grade	0	6	8.6
	I	3	4.3
	II	15	21.4
	III	46	65.7
T stage	1	60	85.7
	2	9	12.9
	3	0	0
	4	1	1.4
N stage	0	49	70
	1	21	30

Supplementary Figure 2. The clinical characteristics of cervical cancer patients. (**A** and **B**) Correlation analyses of KMT2A and VDAC1 protein expression in 102 CIN tissue samples. (**D**–**K**) Correlation of KMT2A and VDAC1 protein expression with different clinicopathologic variables in cervical cancer patient tissues.
