# Supplementary material for: KMT2A regulates cervical cancer cell growth through targeting VDAC1
Source: Aging (Albany NY). 2020 May 21;12(10):9604–20. doi: 10.18632/aging.103229 (PMC7288919; doi:10.18632/aging.103229)
Supplement: Supplementary Table 1 [file aging-12-103229-s002..pdf]

SUPPLEMENTARY TABLE

Supplementary Table 1. The sequences of shRNA and PCR primers.

	Name	Sequence
KMT2A knockdown	KMT2A-shRNA1 forward	5'-CCGGGCACTGTTAAACATTCCACTTCTCG AGAAGTGGAATGTTTAACAGTGCTTTTTG-3'
	KMT2A-shRNA1 reverse	5'-AATTCAAAAAGCACTGTTAAACATTCCAC TTCTCGAGAAGTGGAATGTTTAACAGTGC-3'
	KMT2A-shRNA2 forward	5'-CCGGCCCATCCAGAACCAGAAGTATCTCGA GATACTTCTGGTTCTGGATGGGTTTTTG-3'
	KMT2A-shRNA2 reverse	5'-AATTCAAAAACCCATCCAGAACCAGAAGTAT CTCGAGATACTTCTGGTTCTGGATGGG-3'
qPCR	VDAC1 forward	5'-ACGTATGCCGATCTTGGCAAA -3'
	VDAC1 reverse	5'-TCAGGCCGTACTCAGTCCATC -3'
	GAPDH forward	5'-GGAGCGAGATCCCTCCAAAAT -3'
	GAPDH reverse	5'-GGCTGTTGTCATACTTCTCATGG -3'
